# Exploring Intraspecific Trait Variation in a Xerophytic Moss Species *Indusiella thianschanica* (Ptychomitriaceae) across Environmental Gradients on the Tibetan Plateau

**DOI:** 10.3390/plants13070921

**Published:** 2024-03-22

**Authors:** Xiaotong Song, Jiqi Gu, Yanhui Ye, Mengzhen Wang, Ruihong Wang, Heping Ma, Xiaoming Shao

**Affiliations:** 1 College of Resources and Environmental Sciences, China Agricultural University, Beijing 100193, China; songxt@cau.edu.cn (X.S.); gujq@mail.bnu.edu.cn (J.G.); wangmengzhenl@163.com (M.W.); 2 Beijing Key Laboratory of Biodiversity and Organic Farming, China Agricultural University, Beijing 100193, China; 3 State Key Laboratory of Earth Surface Processes and Resource Ecology, College of Life Sciences, Beijing Normal University, Beijing 100875, China; 4 Resources & Environment College, Tibet Agriculture & Animal Husbandry University, Nyingchi 860000, China; yeyanhui@xza.edu.cn; 5 Institute of Tibet Plateau Ecology, Tibet Agricultural & Animal Husbandry University, Nyingchi 860000, China; wangruihong0511@126.com (R.W.); maheping@xza.edu.cn (H.M.); 6 Key Laboratory of Forest Ecology in Tibet Plateau, Tibet Agricultural & Animal Husbandry University, Ministry of Education, Nyingchi 860000, China

**Keywords:** plant adaptations, morphological traits, phenotypic plasticity, bryophyte ecology, alpine ecosystem

## Abstract

Investigating intraspecific trait variability is crucial for understanding plant adaptation to various environments, yet research on lithophytic mosses in extreme environments remains scarce. This study focuses on *Indusiella thianschanica* Broth. Hal., a unique lithophytic moss species in the extreme environments of the Tibetan Plateau, aiming to uncover its adaptation and response mechanisms to environmental changes. Specimens were collected from 26 sites across elevations ranging from 3642 m to 5528 m, and the relationships between 23 morphological traits and 15 environmental factors were analyzed. Results indicated that coefficients of variation (CV) ranged from 5.91% to 36.11%, with gametophyte height (GH) and basal cell transverse wall thickness (STW) showing the highest and lowest variations, respectively. Temperature, elevation, and potential evapo-transpiration (PET) emerged as primary environmental drivers. Leaf traits, especially those of the leaf sheath, exhibited a more pronounced response to the environment. The traits exhibited apparent covariation in response to environmental challenges and indicated flexible adaptive strategies. This study revealed the adaptation and response patterns of different morphological traits of *I. thianschanica* to environmental changes on the Tibetan Plateau, emphasizing the significant effect of temperature on trait variation. Our findings deepen the understanding of the ecology and adaptive strategies of lithophytic mosses.

## 1. Introduction

Plant functional traits (PFTs) are easily measured plant attributes including morphological, physiological, or phenological characteristics [1,2]. PFTs are closely related to plant ecological functions and reflect plant adaptability and performance in important ecological functions such as resource acquisition, allocation, conservation, and stress response [3,4]. Research on PFTs contributes to a better understanding of plant adaptations and survival strategies, as well as their interactions with the environment [5,6]. Exploring trait variation along environmental gradients is a common approach to elucidating the adaptation of plant functional traits, which can indicate the evolutionary processes of shaping traits and species distributions and predict the species’ adaptability to future climate change [7,8,9]. Although the significant implications of intraspecific trait variation for ecological and evolutionary processes have been well recognized [10,11,12,13], the within-species responses of traits to environmental gradients may vary depending on the traits and species and are hard to predict across traits, species, and locations [14,15,16,17]. Currently, our understanding of intraspecific trait variation is still limited [18].

Intraspecific trait variation is strongly driven by environmental factors, including irradiance, temperature, and aridity [13]. Widely distributed species can exhibit functional trait variation and covariation among traits across environmental gradients, even in environmentally stressed highland and arid ecosystems [19,20,21,22]. The extent and patterns of intraspecific trait variation are critical to understanding the biological traits and ecological significance of different taxa in different environments. Bryophytes are an under-recognized and under-studied group of plants with relatively simple structures that differ significantly from vascular plants in structure and physiology [23], and are often more widely distributed than vascular plant species [24]. Although phylogenetically and structurally distinct from tracheophytes, bryophytes are equally capable of exhibiting trait variation and coordination along environmental gradients [25,26,27]. Examining functional traits in bryophytes can potentially confirm or extend the principles of plant function described for vascular plants [26].

*Indusiella thianschanica* Broth. & Müll. Hal., belonging to the family Ptychomitriaceae, is a typical and interesting small xerophytic moss species. It occurs in environmentally stressful habitats such as arctic-alpine, high-altitude desert, and steppe regions, and is considered a rare species in many regions [28,29]. In China, one of the main habitats of *I. thianschanica* are the high-altitude, cold, and arid regions of the Tibetan Plateau, where it grows on dry exposed rock surfaces or thin soils on rock faces. *I. thianschanica* has unique features that easily distinguish it from other moss species: its bistratose leaves are broadly spirally inrolled above the sheathing base, with the external surface of leaf cells thick-walled, and the internal surface thin-walled [30]. This species shows excellent adaptation to extreme arid environments and plays an important role in ecosystems, especially when often subjected to environmental stresses such as drought.

Within different environmental conditions, intraspecific trait variability is crucial for species establishment, survival, and reproductive capacity. This is particularly true for lithophytic and drought-resistant species, whose morphological trait variability may exhibit uniqueness in adapting to extreme environmental challenges. This study focuses on 26 populations of *I. thianschanica* across varying altitudinal conditions on the Tibetan Plateau, examining the variability of 23 morphological traits. The research objectives were refined into three main aspects: (1) to assess whether intraspecific morphological variability occurs along environmental gradients; (2) to identify key environmental factors closely related to morphological trait variability; and (3) to analyze the interrelationships among these traits and whether covariation exists to address changes in environmental gradients. Through a comprehensive investigation of the relationships between these traits and their association with environmental factors, this study aims to enhance our understanding of the adaptation mechanisms of *I. thianschanica* under the extreme environmental conditions of the Tibetan Plateau.

## 2. Results

### 2.1. Quantitative Analysis of Intraspecific Morphological Variation

In the study of the morphological traits of *I. thianschanica* in Tibet, a significant range of variation was observed across the measured parameters. Coefficients of variation (CVs) ranged from 5.91% to 36.11%, and the phenotypic plasticity indices (PPIs) varied between 0.21 and 0.73 (Table 1). Among all the traits, gametophyte height (GH) exhibited the greatest variation (CV = 36.11%, PPI = 0.73), suggesting a flexible adaptation to environmental conditions. Conversely, transverse wall thickness of basal cells (STW) showed the most stable response to environmental variation among all of the measured traits, with the CV and PPI being the lowest (CV = 5.91%, PPI = 0.21), indicative of a strong morphological resistance to environmental stress and potentially playing a key role in the species’ adaptation to specific habitats.

### 2.2. Relationship between Trait Variation and Environmental Factors

**Figure 1:**
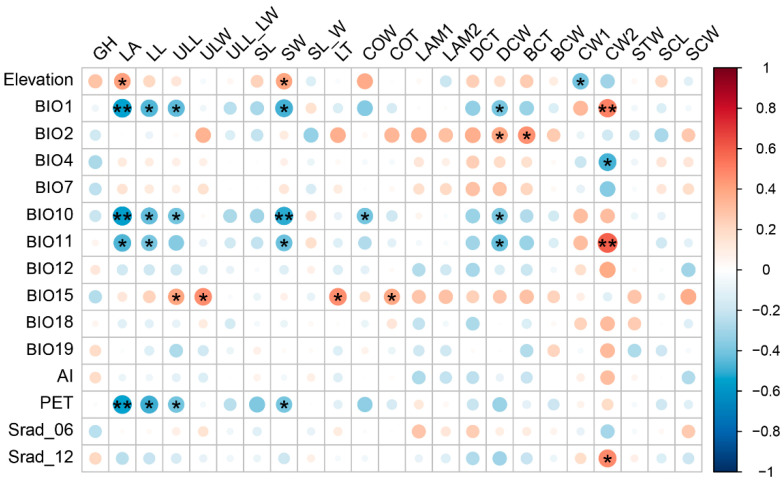
Spearman correlations between environmental factors and morphological traits of *Indusiella thianschanica*. BIO1, annual mean temperature; BIO2, mean diurnal range; BIO4, temperature seasonality; BIO7, temperature annual range; BIO10, mean temperature of the warmest quarter; BIO11, mean temperature of the coldest quarter; BIO12, annual precipitation; BIO15, precipitation seasonality (coefficient of variation); BIO18, precipitation of the warmest quarter; BIO19, precipitation of the coldest quarter; Srad_06, solar radiation in June; Srad_12, solar radiation in December. PET, potential evapo-transpiration; AI, aridity index. Color intensity and size of circles are proportional to the strength of the correlation (red = positive correlation, blue = negative correlation). (*) denotes the level of significance of correlation coefficient: “*” indicates *p* < 0.05, “**” indicates 0.001 < *p* < 0.01.

We examined the correlations between each trait and 15 environmental factors, with the correlation matrix revealing several significant relationships (Figure 1). Specifically, elevation was positively correlated with leaf area (LA) and leaf sheath width (SW), but negatively correlated with thickness of upper surface cell wall (CW1). Annual mean temperature (BIO1), mean temperature of the warmest quarter (BIO10), and mean temperature of the coldest quarter (BIO11) showed negative correlations with LA, leaf length (LL), upper leaf length (ULL), SW, and width of adaxial cell lumen (DCW). BIO1 and BIO11 were positively correlated with thickness of lower surface cell wall (CW2). Mean diurnal range (BIO2) was positively correlated with DCW and thickness of abaxial cell lumen (BCT). Temperature seasonality (BIO 4) was negatively correlated with thickness of lower surface cell wall (CW2). Potential evapo-transpiration (PET) negatively correlated with leaf size traits including LA, LL, ULL and SW. Solar radiation in December (Srad_12) positively correlated with CW2. Precipitation seasonality (BIO15) positively correlated with upper leaf size traits including ULL, ULW, leaf thickness (LT), as well as costa thicknesss (COT). Overall, the temperature factors were more closely related to the traits than the precipitation factors.

**Figure 2:**
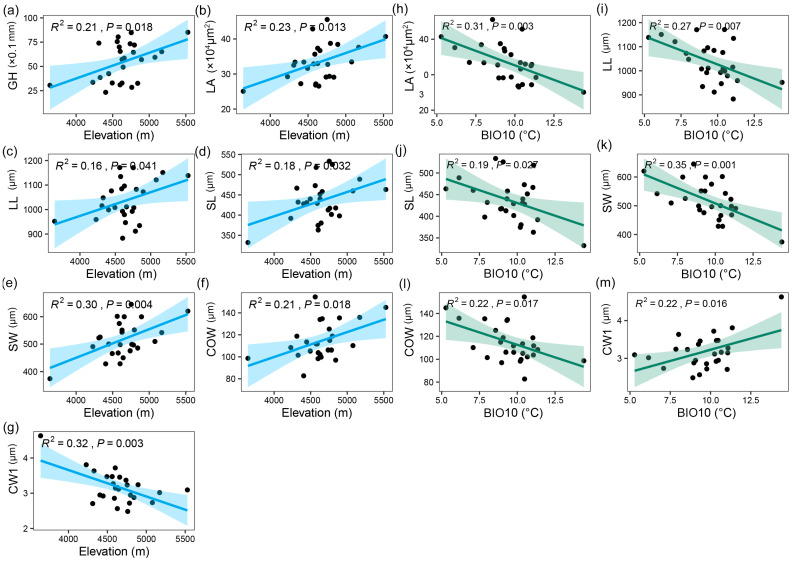
Trait variations of *Indusiella thianschanica* along environmental gradients. The figure illustrates the linear relationships between traits and environmental gradients. Panels (**a**–**g**) represent traits correlated with altitude, including (**a**) gametophyte height (GH), (**b**) leaf area (LA), (**c**) leaf length (LL), (**d**) leaf sheath length (SL), (**e**) leaf sheath width (SW), (**f**) costa width (COW), and (**g**) the thickness of the upper surface cell wall (CW1). Panels (**h**–**m**) depict traits showing a linear relationship with the mean temperature of the warmest quarter (BIO10), including (**h**) leaf area (LA), (**i**) leaf length (LL), (**j**) leaf sheath length (SL), (**k**) leaf sheath width (SW), (**l**) costa width (COW), and (**m**) the thickness of the upper surface cell wall (CW1). The blue or green solid lines in the figures represent the line fitted by a simple linear regression, and the shaded area represents the 95% confidence interval.

Simple linear regression and Loess fit analysis revealed the response patterns of *I. thianschanica* morphological traits along environmental gradients (Figure 2, Figures S1 and S2). Two gradients, elevation and mean temperature of the warmest quarter (BIO10), were selected based on the geoclimatic characteristics of the study area. The results showed that seven traits exhibited significant linear relationships with elevation, with GH, LA, LL, leaf sheath length (SL) and SW, and costa width (COW) increasing with elevation and CW1 decreasing with elevation (Figure 2a–g). Six traits showed significant linear relationships with BIO10, with LA, LL, SL, SW, and COW decreasing with BIO10 and CW1 increasing with increasing BIO10 (Figure 2h–m). Leaf size varied significantly with altitude and temperature gradients, whereas the thickness of the upper surface cell wall was a unique trait, showing the opposite trend to the other traits. Other traits did not show significant linear relationships with the two gradients, suggesting that they were possibly affected by other factors as well.

**Figure 3:**
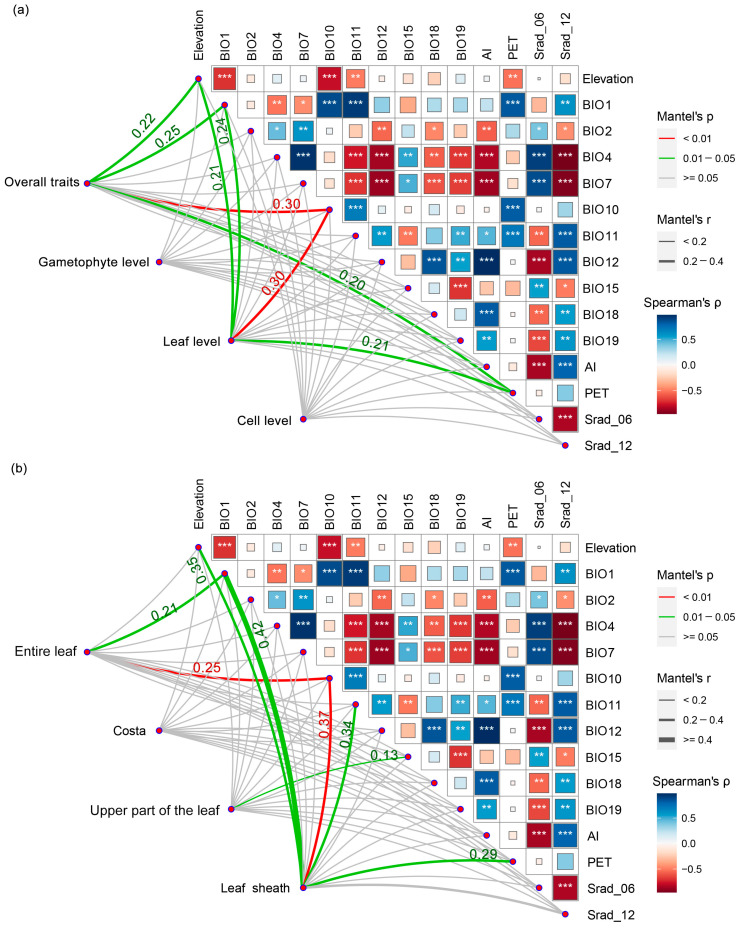
Mantel test for the relationships between different tissue-level traits and environmental factors in *Indusiella thianschanica*. (**a**) Gametophyte-leaf-cell levels and environmental factors. (**b**) Different leaf structures and environmental factors. Mantel’s r (coefficient of correlation) represents the correlation in which the thickness of the line represents the level of correlation and the color of the lines represents the *p*-value. Heatmaps show the Spearman correlation between environmental variables. BIO1, annual mean temperature; BIO2, mean diurnal range; BIO4, temperature seasonality; BIO7, temperature annual range; BIO10, mean temperature of the warmest quarter; BIO11, mean temperature of the coldest quarter; BIO12, annual precipitation; BIO15, precipitation seasonality (coefficient of variation); BIO18, precipitation of the warmest quarter; BIO19, precipitation of the coldest quarter; Srad_06, solar radiation in June; Srad_12, solar radiation in December. PET, potential evapo-transpiration; AI, aridity index. (*) denotes the level of significance of correlation coefficient: “*” indicates *p* < 0.05, “**” indicates *p* < 0.01, and “***” indicates *p* < 0.001.

The Mantel test analysis revealed the relationship between traits and environmental factors at different tissue levels in *I. thianschanica* (Figure 3a). Elevation, BIO1, BIO10, and PET significantly influenced overall traits, with BIO10 having the largest effect. At different tissue levels, from gametophyte to leaf to cell, only the leaf-level traits were significantly affected by these environmental factors, with BIO10 having the largest effect. Further investigation into the association between leaf structural and environmental factors showed that different structures displayed different patterns of relationships with environmental factors (Figure 3b). The entire leaf was affected by BIO1 and BIO10. The upper part of the leaf was only influenced by BIO15, whereas the leaf sheath traits were affected by a combination of factors, including temperature (BIO1, BIO10, BIO11), elevation, and PET, with BIO10 having the most pronounced influence. Costa did not exhibit a significant response. Overall, the results indicated that temperature-related factors had more pronounced effects on traits at different tissue levels as well as leaf structure than precipitation factors.

### 2.3. Interrelationships among Morphological Traits and Trait Covariation

**Figure 4:**
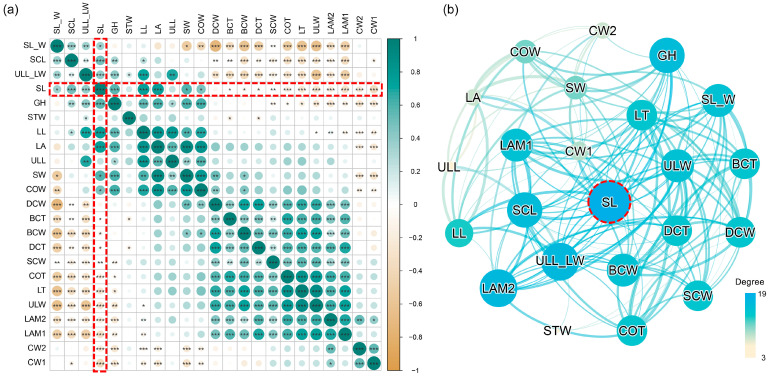
Interrelationships among the 23 morphological traits in *Indusiella thianschanica*. (**a**) The Pearson correlation matrix between the 23 traits. Color intensity and size of circles are proportional to the strength of the correlation (green = positive correlation, brown = negative correlation). (*) denotes the level of significance of the correlation coefficient and “*” indicates *p* < 0.05, “**” indicates *p* < 0.01, “***” indicates *p* < 0.001. (**b**) The network of significantly correlated traits. The thickness of the line is used to indicate the strength of the interrelationship between the nodes, the thicker the line, the greater the correlation. The node size indicates the size of the degree, the larger the node, the larger its degree value, and the more important the node’s position in the network. Red boxes and circle highlight the traits with the highest degree and their correlations with other traits.

Considering the significance of intraspecific variation in morphological traits and their interrelationships for understanding the adaptive strategies of species, we further analyzed and visualized the trait–trait relationships within *I. thianschanica* (Figure 4). The correlation matrix showed complex interconnections between traits, with the correlations between pairs of traits ranging from strong to weak. Significant positive correlations were found within multiple traits, suggesting coordination within traits. Conversely, negative correlations between some traits, which probably reflected trade-offs or differential resource allocation strategies (Figure 4a). The construction of the trait network further visualized the complex relationships among the traits (Figure 4b), and the results showed that among the 23 traits, SL had the highest degree, followed by the ratio of length-to-width of the upper leaf (ULL_LW), and abaxial lamina thickness (LAM2). They were important “hub” traits, with a high degree of interaction with the other traits [31].

**Figure 5:**
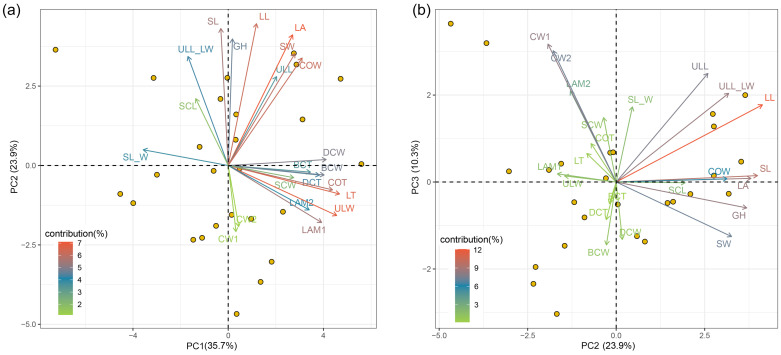
Principal component analysis (PCA) on the 23 traits. Bivariate plots show how each trait is correlated with the first three axes. (**a**) The correlations between the traits and the first two principal axes (PC1 and PC2). (**b**) The correlations between the traits and the second and third principal axes (PC2 and PC3).

The PCA of the 23 morphological traits of *I. thianschanica* illustrated the primary patterns of variation, with the first three principal components (PCs) cumulatively accounting for 69.9% of the total observed variation (Figure 5). PC1 explained 35.7% of trait variation and was strongly influenced by several traits, with LT, ULW, and costa thickness (COT) contributing more to PC1 (Figure S3 and Figure 5a). These traits, indicative of leaf size and structure, showed a concerted positive loading on PC1, suggesting their synergistic relationship in defining the leaf morphology in the species. PC2 explained 23.9% of trait variation, with LL, SL, LA, and GH contributing more to this component (Figure S3 and Figure 5a,b). These traits were related to gametophyte and leaf size, and exhibited a concerted positive loading on PC2, indicating their combined effect on gametophyte development and leaf size variation. The PC3 explained 10.3% of trait variation, mainly contributed by CW1 and CW2 (Figure S3 and Figure 5b). The results of PCA reflected the importance of leaf traits in intraspecific morphological variations of *I. thianschanica* (Figure 5 and Figure S3).

**Figure 6:**
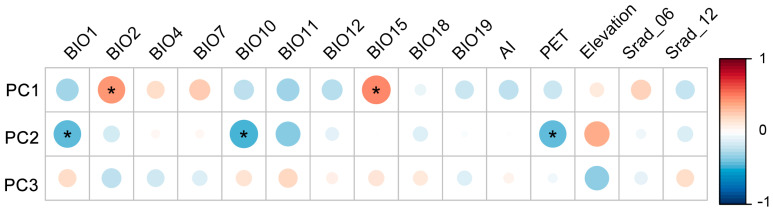
Spearman’s correlation of the first three principal components with environmental factors. Color intensity and size of circles are proportional to the strength of the correlation (red = positive correlation, blue = negative correlation). (*) Denotes the level of significance of correlation coefficient and “*” indicates *p* < 0.05. BIO1, annual mean temperature; BIO2, mean diurnal range; BIO4, temperature seasonality; BIO7, temperature annual range; BIO10, mean temperature of the warmest quarter; BIO11, mean temperature of the coldest quarter; BIO12, annual precipitation; BIO15, precipitation seasonality (coefficient of variation); BIO18, precipitation of the warmest quarter; BIO19, precipitation of the coldest quarter; Srad_06, solar radiation in June; Srad_12, solar radiation in December. PET, potential evapo-transpiration; AI, aridity index. PC1, PC2, and PC3 represent the first, second, and third principal components, respectively.

The correlation analysis of the first three principal components (PC1, PC2, and PC3) with fifteen environmental factors revealed that the variations captured by each PC were influenced by different environmental factors (Figure 6). Specifically, PC1 exhibited significant positive correlations with BIO2 and BIO15, while PC2 exhibited significant negative correlations with BIO1, BIO10, and PET. However, PC3 did not show any significant correlations with the given environmental factors. PC1 was primarily related to the traits of thickness and width of the upper leaves, suggesting that, in environments characterized by greater mean diurnal range and precipitation seasonality, the upper portion of the leaves tends to become thicker and wider. PC2 reflected traits mainly related to the size of the gametophyte and leaf dimensions, indicating that, in colder environments with lower potential evapo-transpiration, *I. thianschanica* tended to exhibit higher gametophytes possessing larger leaf areas, longer leaves, and leaf sheaths.

## 3. Discussion

### 3.1. Intraspecific Morphological Variation across Environmental Gradients

In this study, we examined the morphological variation in *I. thianschanica* from Tibet across environmental gradients and its capacity for morphological adaptation to the extreme and high-altitude conditions. Among the 23 examined morphological traits, GH exhibited high phenotypic plasticity (Table 1), which was consistent with the findings of other moss species in Tibet [25,27]. This finding highlighted the potential role of GH in enhancing the ecological adaptability of moss on the Tibetan Plateau. Leaf-level traits showed some degree of variability (Table 1), which was also attributed to the adaptive response to environmental conditions (Figure 3), indicating the importance of leaf traits in ecological adaptation within species. Variation in cell-level traits may reflect adaptive mechanisms at a microstructural level, with a high plasticity index observed on surface cell walls (Table 1). In contrast, the STW displayed the least variability and was minimally influenced by other traits (Table 1 and Figure 4), suggesting a stability across environmental gradients that might play a key role in helping the species’ adaptation to its unique habitat. Our findings revealed variability in the sensitivity of *I. thianschanica* to environmental change, suggesting that the species may rely on the stability of certain traits to adapt to specific conditions, while utilizing the morphological plasticity of other traits to respond to changing environments.

### 3.2. The Significant Role of Leaf Sheath: From Environmental Response to Species Adaptation

We found that leaf-level traits responded significantly to environmental changes (Figure 3a), with the response of leaf sheath to environmental factors being particularly noteworthy (Figure 3b). In contrast to the upper dark green leaves, the light-colored to transparent leaf sheaths were not the primary photosynthesizing area, but may serve multiple functions; for example, the sheath links the leaf to the stem, providing a certain degree of protection and support. The poikilohydric nature of moss causes the degree of leaf rolling around the stem to vary with water content [32]. The single-layered cell sheaths contribute to rapid water absorption, allowing the leaves to spread and promoting photosynthesis and gas exchange. During drought, the sheaths assist in keeping the leaves close to the stem, slowing water evaporation. Many dominant moss species on the Tibetan Plateau possess similar leaf sheath structures, such as species in the genera *Pogonatum*, *Oncophorus*, and *Distichium*. Previous research has mainly emphasized the role of leaf sheaths in species classification and identification. Our study showed that leaf sheaths have excellent performance in environmental responsiveness and interactions with other traits (Figure 3b and Figure 4), which may contribute to the adaptive capacity of mosses when across environmental gradients.

### 3.3. Key Environmental Factors Driving Intraspecific Trait Variability

The low temperatures and significant temperature fluctuations of the Qinghai–Tibet Plateau pose challenges to plant adaptability [33,34,35]. Our study examined the impact of fifteen environmental variables on the morphological traits of *I. thianschanica* in Tibet. We found that elevation, temperature, and PET significantly influence intraspecific trait variability (Figure 1), indicating morphological adaptation of this species to the unique environmental conditions of the Qinghai–Tibet Plateau. In contrast, precipitation had less effect on the species’ morphological traits. This could be attributed to the long-term evolutionary process, drought-tolerant moss species have adapted to arid environments where precipitation is scarce and have developed a set of survival strategies adapted to arid environments [36]. For instance, specialization in two types of chlorophyllous tissues enables *I. thianschanica* to promote photosynthesis in habitats with only brief periods of moisture during the summer, sometimes relying solely on dew [30]. Our findings further corroborated that temperature, rather than precipitation, was the primary driver of trait variability within bryophyte species in the arid and semi-arid regions of Tibet [25].

### 3.4. Trait Covariation and Adaptation Strategies

Plant environmental adaptability and functional optimization are typically achieved through the comprehensive regulation of multiple traits. In this study, we explored the covariation among morphological traits to reveal the adaptive strategies of *I. thianschanica* to complex environmental gradients (Figure 5 and Figure 6). The positive correlation of PC1 with BIO2 and BIO15 indicated a role for morphological traits of the upper leaf (e.g., thickness and width) in temperature and water regulation [37,38,39]. This morphological adaptation may be a direct response to large diurnal temperature variation or precipitation seasonality. Thicker and wider leaves may assist in the retaining of moisture and heat, thereby enhancing the plant’s tolerance to environmental fluctuations. The negative correlation of PC2 with BIO1, BIO10, and PET was likely to reflect an adaptive strategy to cold and low-potential evapotranspiration environments. Larger gametophytes and leaf area helped this species maximize the use of seasonal precipitation and heat in extreme regions with short growing seasons [37]. The integrated regulation of traits such as gametophyte height and leaf area optimized use of resources to sustain growth and survival in resource-limited environments. The lack of a clear association between PC3 and all environmental factors in this study suggested that the traits captured by PC3 might be influenced by other drivers. In particular, bryophyte cell walls have been proven in the regulation of photosynthetic processes [40,41,42]. Therefore, there may be more complex biological processes involved that need to be further investigated.

## 4. Materials and Methods

### 4.1. Research Site and Plant Material Sampling

**Figure 7:**
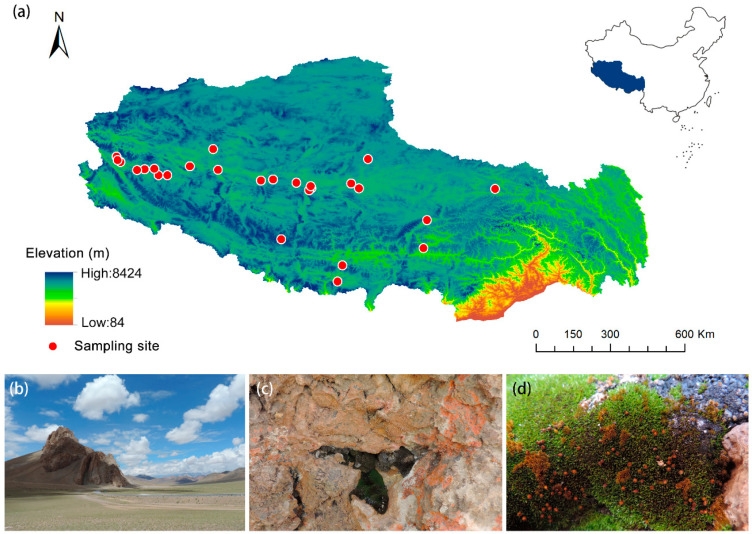
Habitat and sampling sites of *Indusiella thianschanica* in Tibet, China. (**a**) Sampling locations; (**b**) habitat photograph; (**c**) microhabitat photograph; (**d**) ecological habitat photograph.

The study area was located in the Tibet Autonomous Region of China, with an average elevation of over 4000 m. In this study, 26 specimens of *I. thianschanica* were screened and analyzed from 916 specimens of saxicolous bryophytes collected from 244 collection sites. These specimens were distributed across 26 different sites (Figure 7a), and were collected during July and August across the years 2012, 2014, 2019, and 2020. Geographic coordinates and elevations were recorded for each specimen. These 26 sites were located in moderate elevations (3642–5528 m), which belong to the typical arid to semi-arid regions in Tibet. The natural vegetation types included desert steppe, alpine steppe, and alpine meadow. All the specimens were stored in the herbarium of the China Agricultural University (BAU).

**Figure 8:**
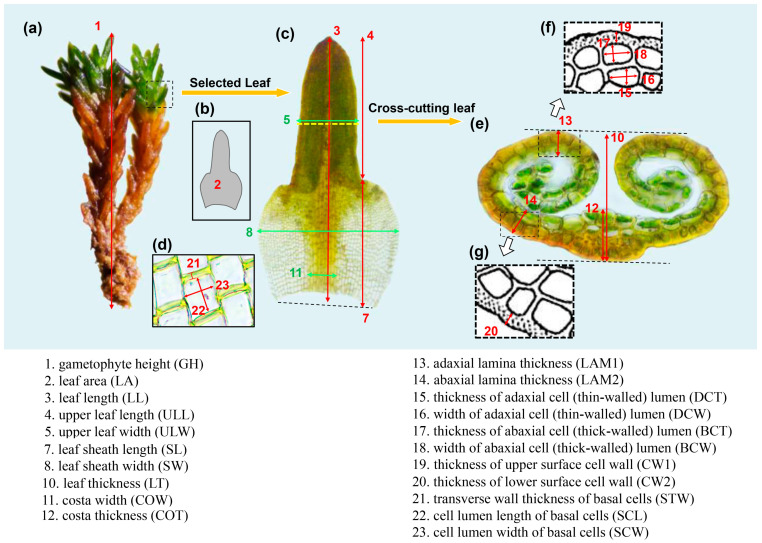
Schematic of the measurement of *Indusiella thianschanica*. (**a**) Gametophyte; (**b**,**c**) leaf; (**d**) leaf sheath cells and (**e**–**g**) cross-section in of the leaf. White arrows point to magnified schematic diagrams of specific sections within the dashed boxes.

*Indusiella thianschanica* occurs in a region featuring a continental climate with wide daily and seasonal temperature fluctuations, intense solar radiation, and extreme dryness for part of the year (Figure 7b). The plants of *I. thianschanica* are densely dark green to black cushions and grow on thin mineral soil on dry rocky surfaces and fissures (Figure 7c,d). The leaves have a sheathing base, and the upper spirally involute lamellae above the base consist of two cell layers, resulting in a succulent appearance (Figure 8c). The ventral cell layer hidden within the involute lamellae is thin-walled and inflated. The dorsal translucent cells are smaller and thick-walled (Figure 8e). The leaf sheath cell is square or rectangular, with straight cell walls, and the transverse cell walls are markedly thickened.

### 4.2. Trait Measurement

Most of the samples we selected had sporophytes, indicating that the gametophytes of the population were well-developed at this point. Ten well-grown plants were selected from each sample to measure their morphological traits [1]. Gametophyte height was measured at natural extension after rehydration (Figure 8a). The morphological traits of the leaves and cells were characterized from whole mounts and cross-sections of one mature leaf from each measured gametophyte sample (Figure 8). In each leaf, measurements were taken from three leaf sheath cells, and the average value was calculated. The leaf was selected from the green leaves in the upper 1/5 of the gametophyte, below which the lower leaves are usually brown. First, the selected intact leaves were placed under a 5× and 40× objective lens of the microscope (AXIO Lab. A1, Carl Zeiss, Jena, Germany) for observation and photography. Then, the leaves were transversely cut with a single-sided blade to obtain cross-sections and observed under a 40× objective. The cross-section was taken from the middle of the spiral involute part of the leaf (Figure 8c,e). Image J software (https://imagej.net/ij/) and ZEN lite software v 3.3 (Carl Zeiss Microscopy GmbH, Jena, Germany) were used to measure the trait values. A total of 23 morphological traits were measured for analysis (Figure 8, Table 2).

### 4.3. Environment Variable

A total of 15 environmental variables were used to explore environment–trait relationships. Ten bioclimatic variables were obtained from WorldClim [43] (http://www.worldclim.org/bioclim, accessed on 30 January 2022), including annual mean temperature (BIO1, °C), mean diurnal range (BIO2, °C), temperature seasonality (BIO4), temperature annual range (BIO7, °C), mean temperature of the warmest quarter (BIO10, °C), mean temperature of the coldest quarter (BIO11, °C), annual precipitation (BIO12, mm), precipitation seasonality (coefficient of variation, BIO15), precipitation of the warmest quarter (BIO18, mm), precipitation of the coldest quarter (BIO19, mm), in addition to two other variables, solar radiation in June (Srad06) and solar radiation in December (Srad12). The data’s spatial resolutions were 30 s (~1 km^2^). The climate-related potential evapo-transpiration (PET) and aridity index (AI) were extracted from Global Aridity and the PET Database [44] (https://csidotinfo.wordpress.com/data/global-aridity-and-pet-database, accessed on 3 March 2023). In addition to this, a topographic factor elevation was included. The geographical coordinates and elevation of each sample location were obtained using a handheld GPS device and the environmental variables data for each sample point were extracted using the latitude and longitude.

### 4.4. Data Analyses

For each sample, the mean of ten measurements for each trait was calculated to represent the trait values of the sample. Subsequently, we calculated the mean, maximum, median, minimum, standard deviation, coefficient of variance (CV), and phenotypic plasticity index (PPI) of each trait across all samples. Spearman’s rank correlation was applied to assess the correlations among environmental variables and the associations between traits and environmental variables. Ordinary linear regression was employed to describe the variation in a single trait response to the environmental gradient, and the 95% confidence intervals were shown. When the relationship between trait and environmental factor was nonlinear, a loess curve was fitted. We used Mantel’s test to analyze the effects of environmental factors on overall traits and on traits at different tissue levels, including gametophyte (cf. Table 2, No. 1)—leaf (cf. Table 2, Nos. 2–14)—cell (cf. Table 2, Nos. 15–23). In addition, we examined the effects of environmental factors on different leaf structures, which included the entire leaf (cf. Table 2, Nos. 2–23), costa (cf. Table 2, Nos. 11, 12), the upper part of leaf (cf. Table 2, Nos. 4–6, 10, 13–20), and the leaf sheath (cf. Table 2, Nos. 7–9, 21–23). Environmental data were scaled and Mantel’s test was performed using the “linkET” package in R (v. 4.2.3). To illuminate the intricate patterns of morphological correlations that exist within the species, a Pearson correlation coefficient matrix was generated and a trait network (TN) was constructed. The trait network was a multi-dimensional network that consisted of nodes and edges. All traits were designated as nodes, while trait–trait relationships were delineated as edges. The strength of the trait–trait relationships was described using absolute Pearson correlations (|r|). A threshold of marked pairwise correlations was used, with *p* < 0.05 considered a significant difference and other relationships were set to zero, yielding the adjacency matrix A = [a_i,j_] with a_i,j_ ∈ [0, 1]. In the trait network, degree was used to quantify the connectedness and importance of each trait, and traits with higher degree were considered to be “hub” traits [31,45]. The trait network was visualized using the software of “Gephi” (https://gephi.org, accessed on 23 March 2023). Finally, the pattern of variation for the 23 traits was analyzed using principal component analysis (PCA) with the R package “vegan”. The relationship between principal components (PCs) and environmental factors was further analyzed using the employed Spearman correlation analysis to identify potential factors affecting multiple correlated traits.

## 5. Conclusions

This study demonstrated the response and morphological adaptations of the lithophytic moss species *I. thianschanica* to extreme environments through detailed analyses of 23 morphological traits at 26 sites in the arid to semi-arid regions on the Qinghai–Tibetan Plateau. We found differences in sensitivity across traits to environmental changes, with leaf sheath playing an essential role in the species’ adaptation and response to extreme environmental conditions. Temperature, elevation, and potential evapotranspiration were identified as key environmental factors driving variation in morphological traits within the species. Additionally, the apparent covariation among traits further revealed the adaptive strategies of this species in response to environmental changes: (1) adapting to habitats with a large mean diurnal range or precipitation seasonality by increasing the width and thickness of the upper part of the leaf, and (2) responding to cold environments with low-potential evapo-transpiration by increasing gametophyte height and leaf area, as well as elongating the leaves and leaf sheaths. This study improved our understanding of species-specific morphological adaptations and provided new insights into plant adaptation to extreme environmental conditions.

## Figures and Tables

**Table 1:** Summary of the morphological traits of *Indusiella thianschanica* in Tibet.

No.	Column	Mean	Median	Maximum	Minimum	SD ^1^	CV ^2^	PPI ^3^
1	GH (×0.1 mm)	54.55	57.84	85.17	23.29	19.70	36.11	0.73
2	LA (×10^4^ μm^2^)	33.32	32.94	45.55	25.06	5.31	15.95	0.45
3	LL (μm)	1036.07	1013.11	1171.30	882.89	82.35	7.95	0.25
4	ULL (μm)	600.39	603.18	698.36	509.89	55.63	9.27	0.27
5	ULW (μm)	214.03	217.49	245.00	175.70	16.38	7.65	0.28
6	ULL_LW	2.82	2.84	3.51	2.32	0.32	11.36	0.34
7	SL (μm)	435.68	431.45	533.43	332.23	49.11	11.27	0.38
8	SW (μm)	517.26	504.85	645.21	374.08	64.93	12.55	0.42
9	SL_W	0.85	0.84	1.11	0.66	0.09	10.76	0.40
10	LT (μm)	115.55	114.74	132.35	97.99	8.27	7.16	0.26
11	COW (μm)	114.36	110.88	154.54	82.69	16.90	14.78	0.46
12	COT (μm)	43.36	42.84	48.82	37.58	3.32	7.67	0.23
13	LAM1 (μm)	22.01	22.20	25.13	17.84	1.67	7.60	0.29
14	LAM2 (μm)	25.72	25.95	28.70	21.50	1.62	6.30	0.25
15	DCT (μm)	11.53	11.72	13.45	8.69	1.11	9.63	0.35
16	DCW (μm)	17.20	17.34	19.35	13.14	1.35	7.83	0.32
17	BCT (μm)	7.42	7.37	8.67	6.22	0.74	9.93	0.28
18	BCW (μm)	9.49	9.36	11.46	7.53	0.89	9.37	0.34
19	CW1 (μm)	3.18	3.13	4.63	2.48	0.46	14.35	0.46
20	CW2 (μm)	4.17	3.90	7.61	2.72	1.08	25.95	0.64
21	STW (μm)	3.58	3.58	3.96	3.14	0.21	5.91	0.21
22	SCL (μm)	15.05	15.16	20.72	11.74	2.11	14.01	0.43
23	SCW (μm)	12.85	12.80	15.20	11.01	0.97	7.56	0.28

^1^ SD: standard deviation. ^2^ CV: coefficient of variation (CV = standard deviation/mean × 100%). ^3^ PPI: phenotypic plasticity index (PPI = (maximum − minimum)/maximum). Values were calculated and rounded to two decimal places for presentation. GH: gametophyte height; LA: leaf area; LL: leaf length; ULL: upper leaf length; ULW: upper leaf width; ULL_LW: ratio of length-to-width of upper leaf; SL: leaf sheath length; SW: leaf sheath width; SL_W: ratio of length-to-width of leaf sheath; LT: leaf thickness; COW: costa width; COT: costa thickness; LAM1: adaxial lamina thickness; LAM2: abaxial lamina thickness; DCT: thickness of adaxial cell (thin-walled) lumen; DCW: width of adaxial cell (thin-walled) lumen; BCT: thickness of abaxial cell (thick-walled) lumen; BCW: width of abaxial cell (thick-walled) lumen; CW1: thickness of upper surface cell wall; CW2: thickness of lower surface cell wall; STW: transverse wall thickness of basal cells; SCL: cell lumen length of basal cells; SCW: cell lumen width of basal cells.

**Table 2:** Trait variables of *Indusiella thianschanica* used in this study.

No.	Abbreviation	Trait Variable	Unit
1	GH	gametophyte height	0.1 mm
2	LA	leaf area	10^4^ μm^2^
3	LL	leaf length	μm
4	ULL	upper leaf length	μm
5	ULW	upper leaf width	μm
6	ULL_LW	ratio of length-to-width of upper leaf (4/5)	/
7	SL	leaf sheath length	μm
8	SW	leaf sheath width	μm
9	SL_W	ratio of length-to-width of leaf sheath (7/8)	/
10	LT	leaf thickness	μm
11	COW	costa width	μm
12	COT	costa thickness	μm
13	LAM1	adaxial lamina thickness	μm
14	LAM2	abaxial lamina thickness	μm
15	DCT	thickness of adaxial cell (thin-walled) lumen	μm
16	DCW	width of adaxial cell (thin-walled) lumen	μm
17	BCT	thickness of abaxial cell (thick-walled) lumen	μm
18	BCW	width of abaxial cell (thick-walled) lumen	μm
19	CW1	thickness of upper surface cell wall	μm
20	CW2	thickness of lower surface cell wall	μm
21	STW	transverse wall thickness of basal cells	μm
22	SCL	cell lumen length of basal cells	μm
23	SCW	cell lumen width of basal cells	μm

## Data Availability

Data are contained within the article and Supplementary Materials.

## Supplementary Materials

The following supporting information can be downloaded at: https://www.mdpi.com/article/10.3390/plants13070921/s1, Figure S1: Linear regression and loess fit analysis of 23 traits of *Indusiella thianschanica* with elevation; Figure S2: Linear regression and loess fit analysis of 23 traits of *Indusiella thianschanica* with the mean temperature of the warmest quarter (BIO 10); Figure S3: Trait contributions to principal component (PC).
