# Supplementary material for: Exploring Intraspecific Trait Variation in a Xerophytic Moss Species Indusiella thianschanica (Ptychomitriaceae) across Environmental Gradients on the Tibetan Plateau
Source: Plants (Basel). 2024 Mar 22;13(7):921. doi: 10.3390/plants13070921 (PMC11013618; doi:10.3390/plants13070921)
Supplement: Supplementary file 1 [file plants-13-00921-s001.zip › plants-2900401-supplementary.pdf]

Exploring Intraspecific Trait Variation in a Xerophytic Moss Species *Indusiella thianschanica* (Ptychomitriaceae) across Environmental Gradients on the Tibetan Plateau

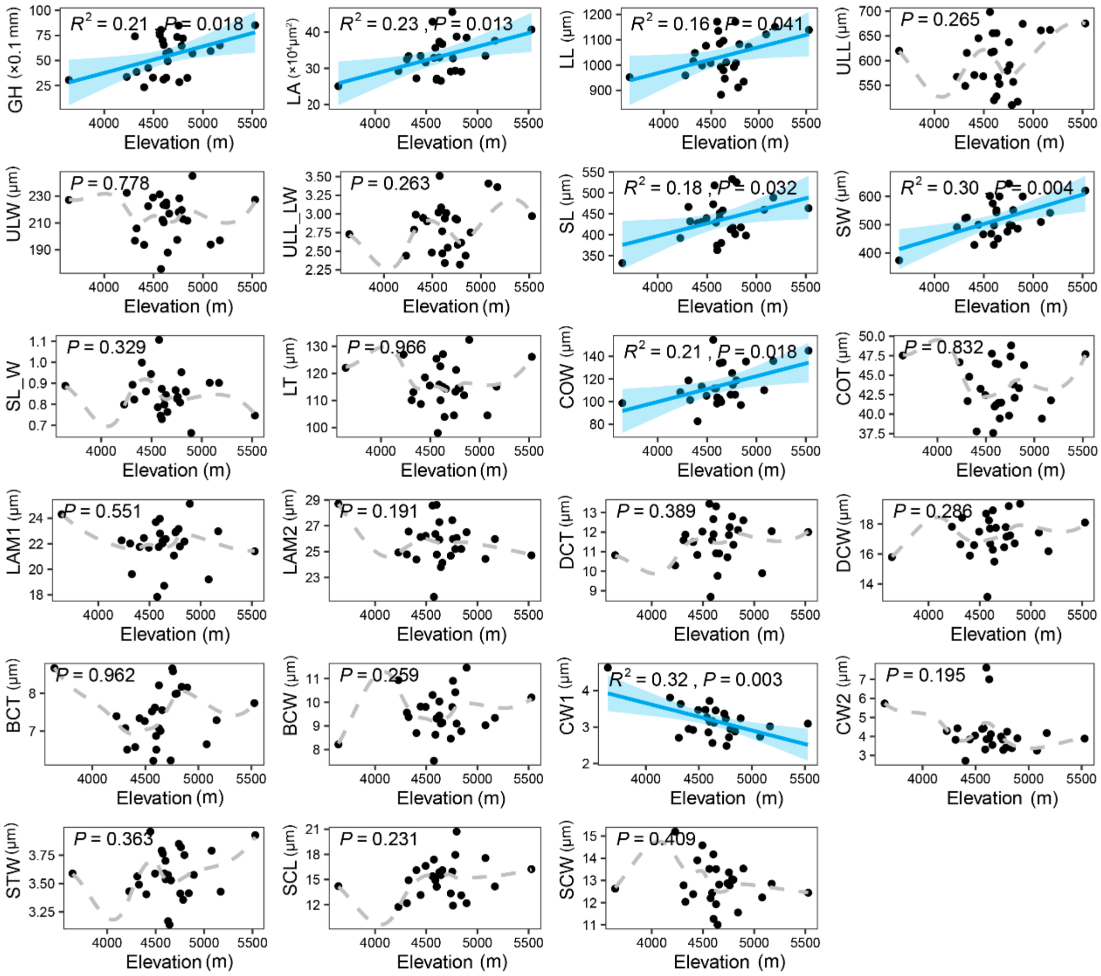

Figure S1. Linear regression and loess fit analysis of 23 traits of *Indusiella thianschanica* with elevation. For a trait that exhibited a linear relationship, linear regression was employed, and 95% confidence intervals were added. For a trait that did not exhibit a linear relationship, a loess curve was fitted. GH: gametophyte height; LA: leaf area; LL: leaf length; ULL: upper leaf length; ULW: upper leaf width; ULL_LW: ratio of length-to-width of upper leaf; SL: leaf sheath length; SW: leaf sheath width; SL_W: ratio of length-to-width of leaf sheath; LT: leaf thickness; COW: costa width; COT: costa thickness; LAM1: adaxial lamina thickness; LAM2: abaxial lamina thickness; DCT: thickness of adaxial cell (thin-walled) lumen; DCW: width of adaxial cell (thin-walled) lumen; BCT: thickness of abaxial cell (thick-walled) lumen; BCW: width of abaxial cell (thick-walled) lumen; CW1: thickness of upper surface cell wall; CW2: thickness of lower surface cell wall; STW: transverse wall thickness of basal cells; SCL: cell lumen length of basal cells; SCW: cell lumen width of basal cell.

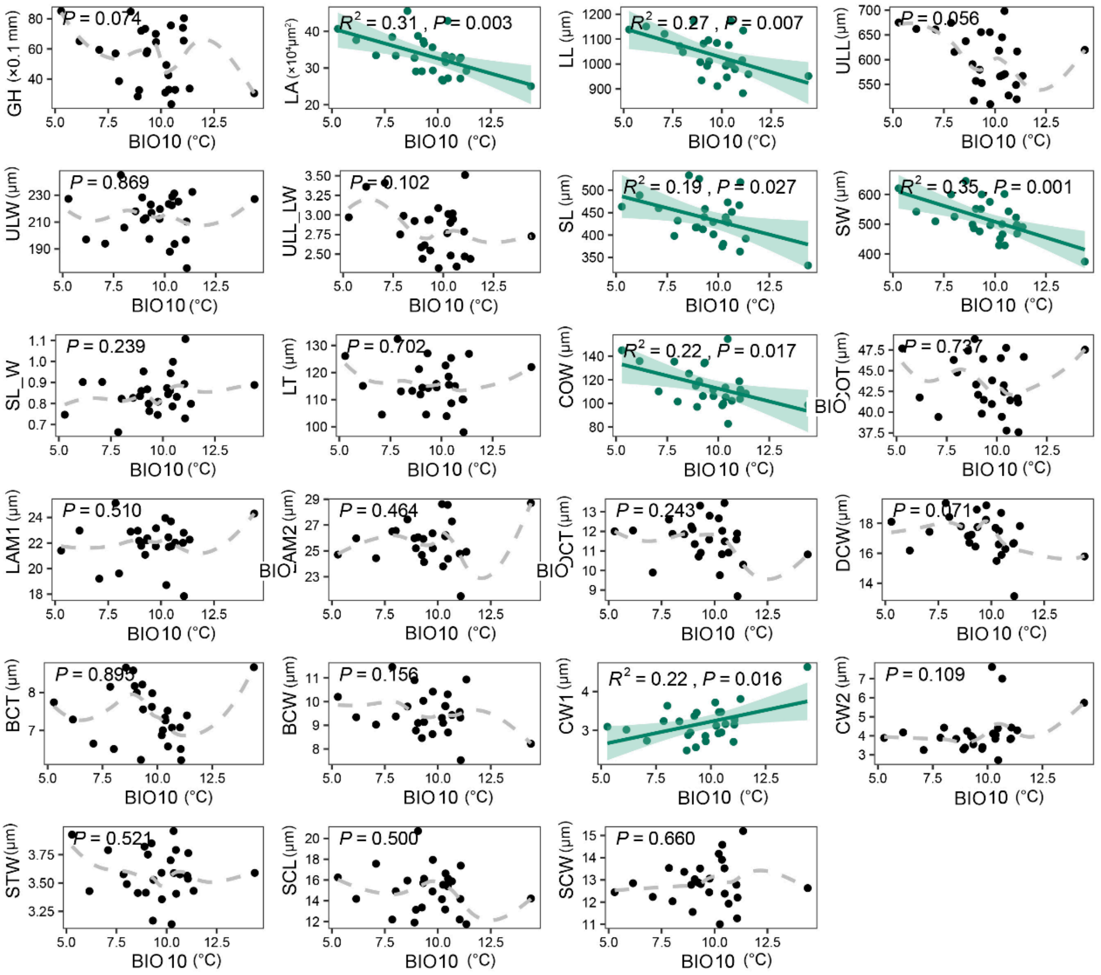

Figure S2. Linear regression and loess fit analysis of 23 traits of *Indusiella thianschanica* with the mean temperature of the warmest quarter (BIO 10). For traits that exhibited a linear relationship, linear regression was employed, and 95% confidence intervals were added. For a trait that exhibited a linear relationship, linear regression was employed, and 95% confidence intervals were added. For a trait that did not exhibit a linear relationship, a loess curve was fitted. GH: gametophyte height; LA: leaf area; LL: leaf length; ULL: upper leaf length; ULW: upper leaf width; ULL_LW: ratio of length-to-width of upper leaf; SL: leaf sheath length; SW: leaf sheath width; SL_W: ratio of length-to-width of leaf sheath; LT: leaf thickness; COW: costa width; COT: costa thickness; LAM1: adaxial lamina thickness; LAM2: abaxial lamina thickness; DCT: thickness of adaxial cell (thin-walled) lumen; DCW: width of adaxial cell (thin-walled) lumen; BCT: thickness of abaxial cell (thick-walled) lumen; BCW: width of abaxial cell (thick-walled) lumen; CW1: thickness of upper surface cell wall; CW2: thickness of lower surface cell wall; STW: transverse wall thickness of basal cells; SCL: cell lumen length of basal cells; SCW: cell lumen width of basal cell.

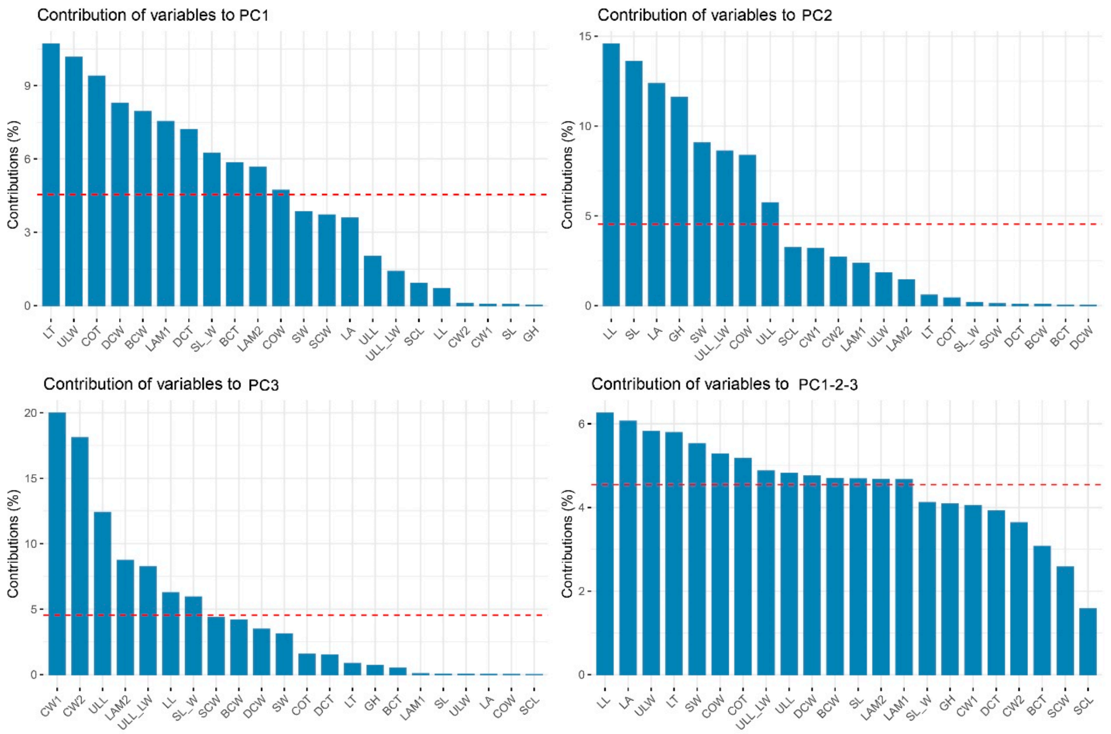

Figure S3. Trait contributions to principal components (PCs). The reference red dashed lines correspond to the expected value if the contributions were uniform.
